# The relationship between blood pressure and cognitive decline differs by race

**DOI:** 10.1093/geront/gnaf189

**Published:** 2025-09-10

**Authors:** Michael D Oliver, Cassandra Morrison, Sondos El-Hulu, Marquinta Harvey, Melissa Lamar, David A Bennett, Lisa L Barnes

**Affiliations:** Department of Psychological Science and Neuroscience, Belmont University, Nashville, Tennessee, United States; Department of Psychology, Carleton University, Ottawa, Ontario, Canada; Department of Psychological Science and Neuroscience, Belmont University, Nashville, Tennessee, United States; Thomas F. Frist, Jr. College of Medicine, Belmont University, Nashville, Tennessee, United States; Department of Public Health, Belmont University, Nashville, Tennessee, United States; Rush Alzheimer’s Disease Center, Rush University Medical Center, Chicago, Illinois, United States; Rush Alzheimer’s Disease Center, Rush University Medical Center, Chicago, Illinois, United States; Rush Alzheimer’s Disease Center, Rush University Medical Center, Chicago, Illinois, United States

**Keywords:** Hypertension, Cognitive change, Racial differences, Variable blood pressure

## Abstract

**Background and Objectives:**

Cognition may be influenced by health-related factors such as blood pressure (BP). However, variations in BP may differentially affect cognition across race. This study investigates BP and cognitive decline in older Black and White adults.

**Research Design and Methods:**

A total of 2,284 participants (1,139 Blacks, 1,145 Whites, *M*_Age_ = 73.4) from three harmonized cohorts of older adults from the Rush Alzheimer’s Disease Center, were divided into three groups (normal, high, variable) based on systolic BP mean and standard deviation. Cognitive scores were computed from 18 neuropsychological tests and averaged to summarize five domains (episodic memory, semantic memory, working memory, processing speed, visuospatial ability) and a measure of global cognition. Linear mixed-effects models examined racial differences between BP and cognition over an average of 6.7 years.

**Results:**

White adults with high BP declined faster in global cognition, perceptual speed, semantic memory, and working memory compared to Black adults with high BP, whereas White adults with variable BP had faster rates of decline in global cognition, all cognitive domains, compared to Black adults with variable BP. No differences in rate of cognitive decline were observed between Black and White older adults with normal BP.

**Discussion and Implications:**

Variations in BP differentially relate to cognitive decline in Black and White older adults, highlighting the interplay between BP and cognitive health, and the importance of race in understanding this relationship.

## Background and objectives

According to the World Health Organization, hypertension is estimated to affect nearly 1.3 billion people worldwide (WHO, 2023). Several studies in older adults have revealed inverse associations between blood pressure (BP) and cognition (Gifford et al., 2013; Li et al., 2022; Sun et al., 2021), and have identified hypertension as a key modifiable risk factor for cognitive decline secondary to Alzheimer’s disease (AD) and related dementias (Lee et al., 2022; Lennon et al., 2019; Xu et al., 2015). However, the extent to which hypertension affects cognitive abilities may differ by race. It has been well-documented that the prevalence of hypertension is higher in Black individuals compared to White individuals (Hertz et al., 2005; Hines et al., 2022), and this increased prevalence contributes to higher cumulative BP levels in Black individuals that may lead to disparities in cardiovascular disease (Wang et al., 2017, 2022) and cognitive function (Mahinrad et al., 2020).

Several explanations have been proposed to conceptualize differences in health outcomes between Black and White individuals, such as fundamental cause theory, intersectionality, life course theory, social determinants of health, and weathering (Mannor & Malcoe, 2022). One common theme associated with each of these theories is the physiological influence of stress. Stress is a ubiquitous experience associated with race, and numerous studies have documented higher levels of reported stress among Black adults due to a lifetime of discrimination, poverty, and fewer opportunities to climb the social ladder (Goosby et al., 2018; Lim et al., 2022). It is plausible that these stressors influence regulatory systems in the body (Engel, 1977) of Black adults; including the endocrine and nervous systems, both of which are associated with the ability to regulate BP (Chopra et al., 2011; Joyner et al., 2008). Furthermore, a stress response patterned by various social determinants of health over the life course such as discrimination, education, and socioeconomic status, may lead to cognitive impairment secondary to increased wear and tear on the body resulting from prolonged exposure to stress and the body’s attempt to adapt to it, or allostatic load (Forrester et al., 2019).

In other words, physiological changes in the body’s regulatory systems as a result of stress, including the regulation of BP (an original marker of allostatic load [Rodriquez et al., 2019]), may provide early indication of other health outcomes to be experienced later in life, like cognitive decline. An example of the inability to regulate BP affecting cognitive decline comes from a pooled cohort analysis of 19,378 participants from five different studies that revealed that higher overall BP was associated with increased cognitive decline, and Black individuals exhibited higher cumulative BP levels, demonstrating significantly faster decline in memory and global cognition compared to White individuals (Levine et al., 2020). Moreover, not only were cumulative BP rates typically higher, but also control rates tended to be significantly lower in Black individuals compared to White individuals (Abrahamowicz et al., 2023; Hertz et al., 2005). These differences in BP values and control may contribute to differences in the way that BP influences cognitive abilities over time in Black individuals compared to White individuals as the body’s regulatory systems may not respond in the same manner. Given that BP represents a key contributor to allostatic load (Rodriquez et al., 2019), investigations into BP as a means of assessing physiological dysregulation across race may prove vital to understanding these distinctions. Although Black individuals tend to report higher cumulative BP, and higher BP is thought to be suggestive of greater cognitive decline, the effects that BP has on specific cognitive abilities stratified by race are unknown.

Previous research suggests the importance of considering both biological and psychosocial risk and protective factors with respect to optimizing cognition and reducing racial/ethnic health disparities (Zahodne, 2021). For example, in a study using the Midlife in the United States dataset, cross-sectional and longitudinal interactions between psychosocial (e.g., friendship quality) and biological factors (e.g., heart rate variability and inflammatory markers) were associated with cognitive function (Payen et al., 2024). However, the findings were specific to White adults, and the study was unable to replicate their findings in Black adults, suggesting a potential race difference in how biological and psychosocial factors interact to impact cognition. Another study using the Washington Heights-Inwood Columbia Aging Project dataset suggested that psychosocial factors such as discrimination and perceived control may explain racial disparities in cognitive aging beyond socioeconomic indicators (Zahodne et al., 2021). However, this study focused on psychosocial factors to explain racial health disparities but did not look at how these factors interact with biological factors to influence cognition. In both samples, they focused solely on global cognition. Much of the existing literature emphasizes global cognition, yet cognitive decline does not occur uniformly across domains (Glisky, 2007). As such, there is a need for longitudinal studies investigating domain-specific relationships in the interaction between biological and psychosocial factors and cognitive decline by race.

In the current study we use a sociologically informed theory, where race is a social construct that serves as a proxy for a number of “social structural processes that shape life chances and experiences” (Mannor & Malcoe, 2022, p. 62). Moreover, we argue that these life chances and experiences have psychological correlates that interact with the body’s regulatory systems to explain racial health disparities via the biopsychosocial model. This model highlights how biological outcomes, such as BP, do not occur in isolation but are shaped by psychological and social factors. In this study, we conceptualize race as a proxy for social stressors patterned by racism and discrimination, healthcare inequities, and other social determinants of health which may cause dysregulated biological outcomes (i.e., BP). We previously reported on the association of high BP and BP variability to incident dementia among predominantly non-Latino White individuals (Mahinrad et al., 2023; Shah et al., 2005). In the present study, we extend that work by examining change in global cognition and multiple cognitive domains as it relates to BP for older Black individuals. Further, we extend our work by examining whether the association between cognitive decline, BP, and BP variability differs by race. By using the biopsychosocial model, we posit that disparities in cognition may be explained by differences in the body’s physiological response to social structural processes patterned by race, which we are choosing to measure as differences in BP and BP variability. Finally, the present study uses three major ­datasets with diverse participants in the same geographical area. This approach helps minimize any potential social and/or geographical differences that could influence findings observed in other studies.

## Research design and methods

### Participants

Data were obtained from three harmonized cohort studies on aging and dementia: Minority Aging Research Study (MARS) (Barnes et al., 2012), Rush Memory and Aging Project (MAP) (Bennett et al., 2018), and African American Clinical Core (Schneider et al., 2009). These studies have identical recruitment techniques, testing procedures, and a large overlap of item-level data collected by RAs trained by the same trainers, allowing for data to be merged seamlessly for analyses. Participants were enrolled in the aforementioned studies on a rolling basis and followed annually. Annual evaluations were conducted face-to-face in participants’ homes and included a medical history, neurological examination, BP measurement and cognitive testing (described later). Written informed consent, as well as a repository consent for data sharing, was obtained from all participants, and each study was approved by the Institutional Review Board at Rush University Medical Center. Secondary analyses included in this paper were approved by the Belmont University Institutional Review Board. More information about each cohort study design can be accessed through the RADC Research Resource Sharing Hub (https://www.radc.rush.edu).

Participant inclusion criteria for this study were as follows: (1) at least 55 years of age at baseline, (2) self-report race as Black or White, (3) at least two visits with completed cognitive assessments, and (4) at least two visits with BP readings measured and recorded. There were 4,419 participants (1,189 Black; 3,230 White) with 32,116 follow-up timepoints meeting these criteria. To ensure that any potential race differences observed were not due to age, sex, education, or cognitive diagnostic differences (i.e., cognitively normal, mild cognitive impairment, or dementia) between the groups, a subset of participants from each group were selected based on matching criteria based on sex, cognitive diagnosis, age, and years of education. A total of 2,284 participants (1,139 Black; 1,145 White) with a total of 14,428 ­follow-up time points were eligible.

### BP readings

Mean systolic BP (SBP) and standard deviation (*SD*) were computed for each individual averaged across all timepoints. That is, each participant’s BP readings across all timepoints were averaged to create a mean SBP for each person. The *SD* was then computed for the entire sample. Participants were then divided into the following three groups based on mean and *SD*: (1) *normal SBP—*mean SBP below 130 and *SD* not more than 1 *SD* away from the sample *SD*, (2) *high* SBP—mean SBP greater than or equal to 130 (as per the current American College of Cardiology/American Heart Association Task Force on Clinical Practice Guidelines) (Whelton et al., 2018) and *SD* not more than 1 *SD* away from sample *SD*, and (3) *variable* S*BP—*SBP *SD* was more than 1 *SD* away from the sample *SD*, regardless of whether BP was normal or high. There were six groups: White—normal SBP (*n *= 396), White—high SBP (*n *= 332), White—variable SBP (*n *= 416), Black—normal BP (*n *= 259), Black—high BP (*n *= 351), Black—variable BP (*n *= 529).

### Cognitive assessment

All participants were administered a battery of 21 neuropsychological tests; 18 of which assessed five cognitive domains (Barnes et al., 2016; Wilson et al., 2002). There were seven tests of episodic memory (immediate and delayed recall of Story A of the Wechsler Memory Scale-Revised; immediate and delayed recall of the East Boston Story; Word List Memory, Recall and Recognition), two tests of semantic memory (Verbal Fluency; Boston Naming), three tests of working memory (Digit Span forward and backward; Digit Ordering), four tests of processing speed (Symbol Digit Modalities Test; Number Comparison; two indices from a modified version of the Stroop Test), and two tests of visuospatial ability (Line Orientation; Progressive Matrices). Composite measures of each domain were used in analyses. To create each composite score, individual raw scores were converted to *z*-scores, using the mean and *SD* from the combined cohort at baseline, and *z*-scores for the relevant tests were averaged. An individual’s standard performance across all 18 of these tests was averaged to create a measure of global cognitive function (Lamar et al., 2020). More information for the specific tests used for each category can be obtained from https://www.radc.rush.edu/.

### Statistical analysis

Analyses were performed using “R” software version 4.0.5 (R Foundation for Statistical Computing, Vienna, Austria). Linear mixed-effects models (“lmer,” package “lme4” in R) were used to examine cognitive change over time. Analyses were completed for global cognition and each cognitive domain (episodic memory, visuospatial abilities, processing speed, semantic memory, and working memory), with the interaction between Time-From-Baseline and Group examined. The first analysis was completed with White normal BP as the reference. Because there were two other White groups to compare the Black groups to, the analyses were repeated using White high BP and White variable BP as the reference. Participant ID was included as a categorical random effect to account for repeated measures of the same participant.

The model also included age at baseline, sex, years of education, and baseline diagnosis (categorical variable contrasting mild cognitive impairment, dementia, and AD against the controls) as covariates. We included these covariates despite having matched participants on them to ensure that group differences were not being driven by these potential confounders for a number of reasons: (a) they have been found to be important predictors of cognitive decline; (b) they have been shown to account for some of the racial differences in cognition; and (c) they all influence BP (i.e., BP changes with age [Pinto, 2007], BP differs by sex [Baumgartner et al., 2025; Ji et al., 2020], differs with education [Loucks et al., 2011], and is associated with risk of dementia [De Heus et al., 2021]).

All models were corrected for multiple comparisons using false discovery rate (FDR) (Benjamini & Hochberg, 1995), due to presence of interdependency between the regression and mixed-effects models. All *p*-values were reported as raw values with significance, then determined by FDR correction. All continuous values were *z*-scored within the population prior to the analyses. For the purposes of this study, we compared each White group to each of the three Black groups. Thus, within race-group comparisons are not reported.

## Results

The total sample of 2,284 older adults (1,800 females) had an average age of 73.4 ± 6.6 (73.2 years for Black; 73.6 years for White) and a mean education of 15.2 ± 3.7 (15.0 years for Black; 15.4 years for White). Participant characteristics by group are presented in Table 1. Supplementary Table 1 (see online supplementary material) provides baseline group differences in the demographic and cognitive variables.

**Table 1.** Demographic table by race and by group.

Variable	Black	White	Black normal SBP	Black high BP	Black variable SBP	White normal SBP	White high BP	White variable SBP
**Sample (females)**	1,139 (79%)	1,145 (78%)	259 (82%)	351 (75%)	529 (81%)	396 (83%)	332 (77%)	416 (74%)
**Baseline diagnosis**								
** NC**	845 (75%)	868 (76%)	194 (75%)	268 (76%)	383 (72%)	306 (77%)	250 (75%)	311 (75%)
** MCI**	258 (22%)	238 (21%)	57 (22%)	73 (21%)	128 (24%)	76 (20%)	74 (22%)	87 (21%)
** AD**	32 (3%)	35 (3%)	8 (3%)	9 (3%)	15 (3%)	12 (3%)	5 (2%)	18 (4%)
** Dementia**	4 (<1%)	5 (<1%)	0 (0%)	1 (<1%)	3 (<1%)	2 (<1%)	3 (1%)	0 (0%)
**Age**	73.2 ± 6.6	73.6 ± 6.6	72.4 ± 6.8	72.7 ± 6.6	74.0 ± 6.3	72.4 ± 6.7	73.8 ± 6.5	74.5 ± 6.5
**Education**	15.0 ± 3.5	15.4 ± 3.8	15.0 ± 3.4	15.0 ± 3.4	15.0 ± 3.5	15.7 ± 3.9	15.5 ± 3.7	14.9 ± 3.8
**Mean SBP**	136.1 ± 15.3	130.6 ± 14.1	119.81 ± 6.7	143.1 ± 12.0	139.5 ± 14.6	118.4 ± 7.7	139.9 ± 10.5	135.1 ± 13.1
**Global cognition**	−0.20 ± 0.76	0.17 ± 0.83	−0.16 ± 0.84	−0.16 ± 0.75	−0.24 ± 0.72	0.23 ± 0.85	0.20 ± 0.78	0.09 ± 0.85
**Episodic memory**	−0.18 ± 0.75	−0.01 ± 0.84	−0.18 ± 0.81	−0.15 ± 0.76	−0.20 ± 0.71	0.06 ± 0.82	−0.01 ± 0.78	−0.08 ± 0.90
**Visuospatial ability**	−0.35 ± 0.97	0.24 ± 0.89	−0.31 ± 1.04	−0.27 ± 0.95	−0.42 ± 0.95	0.30 ± 0.98	0.28 ± 0.81	0.16 ± 0.88
**Processing speed**	−0.08 ± 0.75	0.30 ± 0.91	0.01 ± 0.81	−0.07 ± 0.76	−0.14 ± 0.72	0.35 ± 0.96	0.34 ± 0.89	0.22 ± 0.90
**Semantic memory**	−0.15 ± 0.86	0.23 ± 0.81	−0.12 ± 0.94	−0.10 ± 0.88	−0.20 ± 0.81	0.27 ± 0.84	0.22 ± 0.75	0.19 ± 0.82
**Working memory**	−0.14 ± 0.99	0.17 ± 0.99	−0.17 ± 0.93	−0.11 ± 0.90	−0.15 ± 0.86	0.16 ± 0.99	0.26 ± 1.01	0.10 ± 0.98

*Note.* Cognitive scores represent the averaged baseline *z*-scores by group. Sample = total sample size (percentage of females); mean BP = mean BP at all measurement time points; BP = blood pressure; SBP = systolic blood pressure; NC = normal controls; MCI = mild cognitive impairment; AD = Alzheimer’s disease.

### Cognitive change

**Figure 1.**
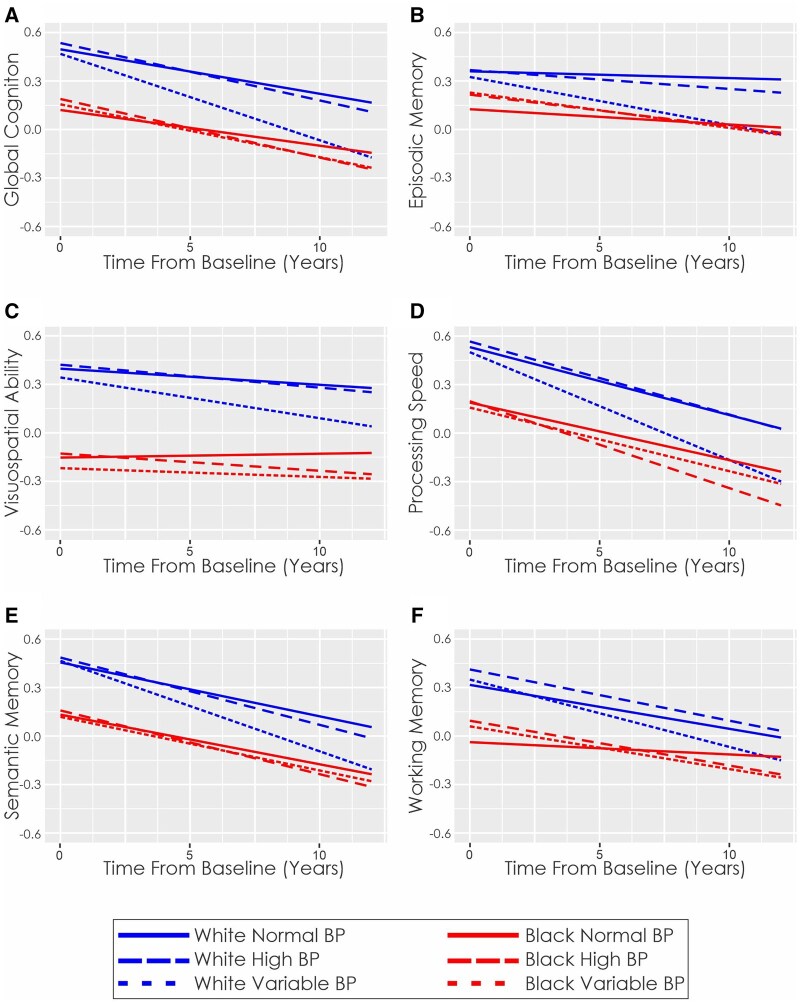
Cognitive change over time by race and blood pressure. This figure demonstrates the predicted longitudinal change in White and Black adults with normal, high, and variable blood pressure across six domains: (A) global cognition, (B) episodic memory, (C) visuospatial ability, (D) processing speed, (E) semantic memory, and (F) working memory. Plotted cognitive scores are *z*-scored values. BP = blood pressure.

The average follow-up for Blacks and Whites was 6.6 ± 4.4 and 6.7 ± 5.4 years (*p* = .42), respectively. Figure 1 shows the mixed-effects model predictions of cognitive scores over time for each cognitive domain by race and BP group. As expected, time from baseline (*p* < .001) and higher age at baseline (*p* < .001) were associated with a faster rate of cognitive change for global cognition, and all cognitive domains (episodic memory, visuospatial abilities, processing speed, semantic memory, and working memory). Table 2 shows the linear mixed-effects model outputs for global cognition and each cognitive domain by race and BP group. All results remained significant after FDR correction.

**Table 2.** Linear mixed-effects models outputs.

Variable	Global cognition	Episodic memory	Visuospatial orientation	Processing speed	Semantic memory	Working memory
**White normal BP as reference**						
** Age**	β = −0.25	β = −0.25	β = −0.12	β = −0.24	β = −0.21	β = −0.09
	*t *= −18.42	*t *= −17.74	*t *= −8.46	*t *= −17.08	*t *= −14.17	*t *= −4.86
	***p* < .001**	***p* < .001**	***p* < .001**	***p* < .001**	***p* < .001**	***p* < .001**
** Male sex**	β = −0.12	β = −0.18	β = 0.26	β = −0.21	β = −0.01	β = −0.04
	*t *= −3.64	*t *= −5.45	*t *= 7.56	*t *= −6.25	*t *= −0.40	*t *= −1.11
	***p* < .001**	***p* < .001**	***p* < .001**	***p* < .001**	*p* = .69	*p* = .26
** Education**	β = 0.26	β = 0.16	β = 0.24	β = 0.23	β = 0.21	β = 0.28
	*t *= 19.55	*t *= 12.00	*t *= 17.12	*t *= 16.60	*t *= 14.11	*t *= 17.24
	***p* < .001**	***p* < .001**	***p* < .001**	***p* < .001**	***p* < .001**	***p* < .001**
** MCI**	β = −0.79	β = −0.80	β = −0.63	β = −0.51	β = −0.64	β = −0.48
	*t *= −23.79	*t *= −23.41	*t *= −17.97	*t *= −14.40	*t *= −17.36	*t *= −11.70
	***p* < .001**	***p* < .001**	***p* < .001**	***p* < .001**	***p* < .001**	***p* < .001**
** Dementia**	β = −1.96	β = −1.85	β = −1.13	β = −0.91	β = −1.77	β = −1.31
	*t *= −25.54	*t *= −25.46	*t *= −13.96	*t *= −11.22	*t *= −20.82	*t *= −13.87
	***p* < .001**	***p* < .001**	***p* < .001**	***p* < .001**	***p* < .001**	***p* < .001**
** Follow-up year**	β = −0.12	β = −0.02	β = −0.04	β = −0.19	β = −0.15	β = −0.12
	*t *= −9.17	*t *= −6.13	*t *= −2.45	*t *= −13.83	*t *= −9.51	*t *= −7.62
	***p* < .001**	***p* < .001**	***p* = .01**	***p* < .001**	***p* < .001**	***p* < .001**
** Black normal BP**	β = −0.36	β = −0.26	β = −0.49	β = −0.32	β = −0.31	β = −0.26
	*t *= −6.86	*t *= −4.94	*t *= −9.06	*t *= −5.78	*t *= −5.46	*t *= −4.17
	***p* < .001**	***p* < .001**	***p* < .001**	***p* < .001**	***p* < .001**	***p* < .001**
** Black high BP**	β = −0.35	β = −0.22	β = −0.53	β = −0.39	β = −0.33	β = −0.22
	*t *= −7.35	*t *= −4.45	*t *= −10.41	*t *= −7.78	*t *= −6.16	*t *= −3.85
	***p* < .001**	***p* < .001**	***p* < .001**	***p* < .001**	***p* < .001**	***p* < .001**
** Black variable BP**	β = −0.36	β = −0.22	β = −0.59	β = −0.36	β = −0.34	β = −0.25
	*t *= −8.57	*t *= −4.92	*t *= −13.13	*t *= −8.02	*t *= −7.09	*t *= −4.83
	***p* < .001**	***p* < .001**	***p* < .001**	***p* < .001**	***p* < .001**	***p* < .001**
** Follow-up × Black normal BP**	β = 0.04	β = 0.02	β = 0.05	β = −0.03	β = 0.01	β = 0.09
	*t *= 1.13	*t *= −0.96	*t *= 1.91	*t *= 1.32	*t *= 0.46	*t *= 3.45
	*p* = .04	*p* = .35	*p* = .06	*p* = .18	*p* = .65	***p* < .001**
** Follow-up × Black high BP**	β = 0.01	β = −0.07	β = 0.01	β = −0.05	β = 0.03	β = −0.01
	*t *= −1.84	*t *= −2.86	*t *= −0.11	*t *= −2.43	*t *= −1.11	*t *= −0.10
	*p* = .53	***p* = .004**	*p* = .86	***p* = .01**	*p* = .27	*p* = .92
** Follow-up × Black variable BP**	β = 0.02	β = −0.09	β = 0.02	β = −0.01	β = 0.01	β = 0.01
	*t *= −1.33	*t *= −3.96	*t *= 0.86	*t *= 0.68	*t *= 0.02	*t *= 0.16
	*p* = .12	***p* < .001**	*p* = .39	*p* = .49	*p* = .97	*p* = .87
**White high BP as reference**						
** Follow-up × Black normal BP**	β = 0.08	β = 0.01	β = 0.07	β = 0.05	β = 0.08	β = 0.15
	*t *= 3.89	*t *= 0.38	*t *= 2.42	*t *= 3.00	*t *= 3.93	*t *= 4.02
	***p* < .001**	*p* = .70	***p* = .015**	***p* = .003**	***p* < .001**	***p* < .001**
** Follow-up × Black high BP**	β = 0.06	β = 0.04	β = 0.02	β = 0.04	β = 0.08	β = 0.06
	*t *= 2.66	*t *= 1.39	*t *= 0.52	*t *= 3.09	*t *= 3.49	*t *= 2.57
	***p* = .007**	*p* = .16	*p* = .60	***p* = .020**	***p* < .001**	***p* = .010**
** Follow-up × Black variable BP**	β = 0.02	β = −0.05	β = 0.04	β = 0.03	β = 0.03	β = 0.07
	*t *= 1.31	*t *= −2.10	*t *= 1.52	*t *= 1.37	*t *= 1.54	*t *= 1.04
	*p* = .19	*p* = .036	*p* = .12	*p* = .17	*p* = .12	*p* = .29
**White variable BP as reference**						
** Follow-up × Black normal BP**	β = 0.14	β = 0.12	β = 0.12	β = 0.14	β = 0.11	β = 0.15
	*t *= 6.81	*t *= 3.82	*t *= 4.42	*t *= 6.65	*t *= 4.68	*t *= 6.22
	***p* < .001**	***p* < .001**	***p* < .001**	***p* < .001**	***p* < .001**	***p* < .001**
** Follow-up × Black high BP**	β = 0.08	β = 0.06	β = 0.06	β = 0.06	β = 0.07	β = 0.06
	*t *= 3.77	*t *= 2.99	*t *= 2.36	*t *= 2.80	*t *= 3.09	*t *= 2.57
	***p* < .001**	***p* = .002**	***p* = .019**	***p* = .005**	***p* = .001**	***p* = .01**
** Follow-up × Black variable BP**	β = 0.09	β = 0.05	β = 0.09	β = 0.12	β = 0.10	β = 0.07
	*t *= 5.62	*t *= 3.13	*t *= 3.94	*t *= 7.31	*t *= 5.27	*t *= 3.46
	***p* < .001**	***p* = .002**	***p* < .001**	***p* < .001**	***p* < .001**	***p* < .001**

*Note*. This table shows the linear mixed-effects model outputs for global cognition and each cognitive domain by race and BP group. Bolded values are those that remained significant after correction for multiple comparisons. BP = blood pressure; MCI = mild cognitive impairment.

#### Race and normal BP

The interaction between follow-up time from baseline and racial group showed that the rate of cognitive decline in White older adults with normal BP did not differ from Black older adults with normal BP on global cognition (*t *= 1.13, *p* = .04),^1^ episodic memory (*t *=* −*0.96, *p* = .35), visuospatial abilities (*t *= 1.91, *p* = .06), processing speed (*t *= 1.32, *p* = .18), or semantic memory (*t *= 0.46, *p* = .65). However, White older adults with normal BP did exhibit an increased rate of decline in working memory compared to their Black counterparts with normal BP (*t *= 3.45, *p* < .001).

#### Race and high BP

White older adults with high BP exhibited significantly greater rates of decline when compared to Black older adults with normal BP in all domains except episodic memory (*t *= 0.38, *p* = .70). However, the rate of decline in global cognition, visuospatial orientation, semantic memory, and working memory was comparable in Blacks with high BP and Whites with normal BP (*p* > .05). Blacks with high BP only had increased decline compared to Whites with normal BP in episodic memory (*t *=* −*2.86, *p* = .004) and processing speed (*t *=* −*2.43, *p* = .01). Additionally, White older adults with high BP had significantly greater rates of decline in global cognition, and all other cognitive domains, except episodic memory (*t *= 1.39, *p* = .16) and visuospatial orientation (*t *= 0.52, *p* = .60) when compared to Black older adults with high BP.

We considered whether medication differences may have impacted results. Supplementary Table 2 (see online supplementary material) includes frequency information about antihypertensive/diuretic use by group. It is important to note that the number of individuals on medication either at baseline or at any point during the study did not differ between groups (i.e., Normal BP Black vs. Normal BP White, High BP Black vs. High BP White, Variable BP Black vs. Variable BP White); therefore, we did not adjust for medication use in analyses.

#### Race and variable BP

For White older adults with variable BP, the rate of cognitive decline in all five domain-specific cognitive abilities (episodic memory, visuospatial abilities, processing speed, semantic memory, working memory), as well as global cognition, was faster compared to Black older adults with normal, high, or variable BP. Conversely, Black older adults with variable BP only exhibited a faster rate of cognitive change in episodic memory when compared to White older adults with normal BP (*t *=* −*3.96, *p* < .001). There were no other differences in rates of cognitive change between Black older adults with variable BP and White older adults with either normal or high BP.

### Additional analyses to test for confounding effects

To ensure results were not driven by the study cohort effects, we repeated all analyses with study cohort as a random categorical effect. For a more conservative assessment, we also repeated the models including Time From Baseline as a random slope. In both analyses, all results remained the same in terms of effect size and significance (Supplementary Table 3, see online supplementary material).

One possible explanation for Black older adults having slower rates of decline is associated with lower cognitive scores at baseline compared to White older adults. To test this possibility, we repeated all analyses including baseline cognitive scores for each domain in the appropriate linear mixed effects models. We observed that the results did not significantly differ in terms of effect size or significance from the original outputs indicating that the differences in groups are not driven by lower baseline cognitive scores in the Black older adults. A secondary analysis was also conducted by removing the 139 (*n *= 62 Black, *n *= 77 White) participants under the age of 65. Results remained similar in terms of effect size and significance.

## Discussion and implications

We investigated the effects of BP on cognitive decline by race. Specifically, we examined relationships between normal, high, and variable BP on global and domain-specific cognitive decline in older Black and White adults. Findings from this study reveal no racial differences in the rate of cognitive decline among individuals with normal BP. White adults with high or variable BP exhibited significantly faster rates of decline in global cognition compared to their Black counterparts. More specifically, White adults with high BP declined faster in perceptual speed, semantic memory, and working memory compared to Black adults with high BP, whereas White adults with variable BP had significantly faster rates of decline in all five cognitive domains when compared to Black adults with variable BP.

Our findings suggest that in individuals with normal BP, the influence of BP on rate of cognitive decline does not differ between Black and White older adults. We should note that in a separate study, race differences in cognitive performance were found to be no longer significant when BP was controlled for in their analyses (Levine et al., 2020). Taken together, when BP is within normal range, race does not appear to be a strong independent factor influencing longitudinal cognitive abilities.

Alternatively, when BP is relatively high, there is a possible compounding effect of this added physiological stressor on long-term cognitive health as explained by biopsychosocial theory. Concordantly, the present study revealed that high BP was associated with greater longitudinal cognitive decline compared to normal BP that was both domain-specific, as well as for global cognition. Elevated BP is a significant risk factor for several health conditions, including those affecting cognitive abilities such as AD and related dementias (Fuchs & Whelton, 2020). High BP has been linked to structural brain changes on magnetic resonance imaging (MRI) associated with cognitive deficits in executive function, processing speed, and learning (Siedlinski et al., 2023). In the present study, in individuals with high BP, older Black and White adults had similar rates of decline in episodic memory and visuospatial abilities, but different rates of decline in global cognition, perceptual speed, semantic memory, and working memory; all of which White individuals exhibited significantly faster rates of cognitive decline compared to Black individuals. Our findings are consistent with several studies suggesting a greater association between high BP and both global cognitive decline (Bohannon et al., 2002; Gottesman et al., 2014), as well as executive function decline (Levine et al., 2020) in White adults compared to Black adults. Therefore, when BP was equal between racial groups, with both Black and White adults having high BP, it appears that high BP affects global cognition, and several domain-specific cognitive abilities more in White older adults than Black older adults over time. Moreover, White older adults with variable BP had significantly faster rates of decline in global cognitive abilities, and all domain-specific cognitive abilities (i.e., episodic memory, visuospatial abilities, perceptual speed, semantic memory, working memory), compared to Black older adults with variable BP. Therefore, although we see similar effects of BP on cognitive abilities to those observed previously (Siedlinski et al., 2023), the deleterious effects of high and variable BP are found to differentially impact cognitive performance over time in White compared to Black older adults.

One potential explanation for our findings can be understood by establishing parallels with the concept of cognitive reserve. Cognitive reserve can be defined as individual differences in experiences or exposures that buffer the brain against cognitive impairment despite damage through aging and/or disease (Stern, 2002, 2012). It has also recently been revealed that cognitive reserve differs as a function of race (Avila et al., 2021). Historically speaking, it has been established that structural racism and discrimination have led to less opportunities for Black individuals compared to White individuals (Braveman et al., 2022). These social disadvantages have resulted in less educational opportunities, job opportunities, and the ability to participate in leisurely activities—all of which are considered proxies for cognitive reserve (Pettigrew & Soldan, 2019). Moreover, these disadvantages significantly reduced opportunities to engage in activities that are now considered to be protective against cognitive decline. As such, Black older adults tend to have less cognitive reserve compared to White older adults, which may also be a potential explanation for why Black individuals tend to have lower baseline cognition compared to White individuals. From a longitudinal standpoint, previous literature reveals that individuals with less reserve not only have lower baseline cognition but also decline at a slower rate (after dementia onset) compared to their counterparts with higher reserve (Meng & D’arcy, 2012; Soldan et al., 2017). Although all participants in the present study were considered cognitively unimpaired with no diagnosis of dementia, concordantly, in the present study, we observed a similar trend as older Black adults not only had lower baseline cognition compared to their White counterparts but also decline at a slower rate compared to older White adults. More specifically, Black older adults with high or variable BP experienced a slower rate of cognitive decline compared to older White adults with high or variable BP. However, this was not the case when BP was normal.

Another potential explanation for the discrepancies in BP’s effect on longitudinal cognitive abilities found in the present study may be the functional status of the autonomic nervous system (ANS)—the branch of the peripheral nervous system that regulates BP. For example, the sympathetic branch of the ANS is dominant as BP is elevated causing blood vessels to vasoconstrict which increases BP. Overactive sympathetic nervous system (SNS) activation is thought to be indicative of prolonged increases in allostatic load—the cumulative “wear and tear” of chronic stress and life events (Zakrzewska-­Pniewska et al., 2012); ultimately leading to sustained high BP (Ayada et al., 2015). Higher allostatic loads and chronic stress are associated with key indicators of accelerated aging (Sin et al., 2015), psychological well-being, and slow deterioration of cognitive functioning (Knight et al., 2020; Seeman et al., 2001; Turner et al., 2017). Moreover, ANS function is associated with cognitive performance (Forte et al., 2019; Hu et al., 2023) irrespective of race. However, it is possible that continuous prolonged activation of the SNS due to various life course social factors may result in a diminished or weakened response to increases in allostatic load (Walsemann et al., 2022). For example, it has been suggested that although Black individuals may exhibit increased SNS activity at rest, they may also display increased heart rate variability compared to White adults, potentially reflecting a sustained compensatory response to adverse stressors (Kemp et al., 2016). This compensatory response may weaken the effect that BP has on cognition. As such, greater lifetime exposures to allostatic load factors, such as racism-­related stress and discrimination (Barnes et al., 2004; Pager & Shepherd, 2008), may result in older Black adults being less affected cognitively by stress-induced physiological changes like elevated BP compared to older White adults.

Although the current study is novel in that it identifies race differences in the relationship between BP and cognition, there are a couple of limitations to note. First, the present study operationalized BP into three distinct categories based on individual yearly means and *SD*s compared to the entire sample. Although this technique provides an objective measure of BP that can be compared across the sample, it does not consider factors such as adherence to medication, which may influence BP. For example, it has been well-documented that adherence to pharmacologic treatment of BP can lead to more controlled and/or reduced BP levels compared to low or no adherence (Hill et al., 2011; Matsumura et al., 2013). A comprehensive history of medication use was not included in the present study as it was beyond the scope of the current study. Although the proportion of individuals taking antihypertensive/diuretic drugs did not statistically differ by race in the present study, future studies may wish to assess medication adherence as a potential covariate in the relationship between BP and cognition. Finally, although the present study includes a large diverse sample obtained from three major datasets within the same geographical location, participants came from a midwestern urban city in the United States and results may not be generalizable to older adults from other parts of the country and/or world. Future studies may wish to replicate this study with participants from various cultural and geographic backgrounds.

It is important to note that the findings of the present study do not refute the role of racial differences in cognitive function. Concordant with extant literature investigating the relationship between race and cognitive health, older Black adults have lower baseline cognitive performance compared to older White adults. However, in the present study, and consistent with previous studies in this cohort (Weuve et al., 2018), we do not observe differences in the rate of cognitive decline when BP is within normal limits. Similarly, race was found to be unrelated to rate of cognitive decline in a 12-year longitudinal study (Castora-Binkley et al., 2015). Race is a social construct; thus, it is likely that social factors associated with race such as inequities in healthcare and quality of education influence cognition in Black adults (Barnes, 2022). For example, many studies have historically described race using sociological theories including intersectionality and/or social determinants of health and have highlighted how sociopolitical processes may contribute to social hierarchies (Mannor & Malcoe, 2022). Social hierarchies may provide cognitive benefits such as ease and control (Zitek & Phillips, 2020). However, these benefits may differ by race. For example, given that Black individuals are disadvantaged in society with regards to educational and occupational opportunities, housing, and access to healthcare, it has been suggested that Black individuals are seen as being lower on the social hierarchy compared to White individuals (Gans, 2012). As such, social influences on health, paired with the psychological toll of coping with societal factors may play a large role in contributing to racial health disparities. Viewing race through a sociologically informed lens may prove vital to our understanding of racial health disparities as the biopsychosocial model provides insight into biological reactivity of regulatory systems in the body in response to chronic stress related to socioeconomic, social, and psychological factors through which race serves as a proxy for varied life experiences. In this sense, we use the model to clarify that the observed racial disparities in BP and cognition are not assumed to be innate or biologically determined, but rather shaped by broader social contexts. Further, our utilization of three harmonized cohort studies within the same geographical location allows for the assumption of similar social conditions, which further supports our conclusion that racial disparities in BP and cognition are shaped by social factors. Nonetheless, our findings highlight that older White adults exhibit a higher susceptibility to cognitive decline when facing elevated and variable BP levels, as compared to their Black counterparts. These findings may be due in part to the fact that prolonged exposure to such inequities, as seen in older Black adults, may mute the physiological impact of BP on cognition. These results shed light on the complex interplay between BP and cognitive health, emphasizing the need to examine race-specific relationships between BP and cognitive performance over time.

## Supplementary Material

gnaf189_Supplementary_Data

## Data Availability

Data used in the present study are publicly available through the RADC Research Resource Sharing Hub. To obtain data from MARS, AA Core, and MAP for research use, please visit the RADC Research Resource Sharing Hub (www.radc.rush.edu). This study was not preregistered.

## Footnotes

1 No longer significant after FDR correction.
