# Supplementary material for: The relationship between blood pressure and cognitive decline differs by race
Source: Gerontologist. 2025 Sep 10;65(12):gnaf189. doi: 10.1093/geront/gnaf189 (PMC12681357; doi:10.1093/geront/gnaf189)
Supplement: gnaf189_Supplementary_Data [file gnaf189_supplementary_data.zip › Oliver et al. Suppl.docx]

**Supplementary Material**

**Supplemental Table 1**

*Group Differences in Demographic and Cognitive Variables*

| Variable | Kruskal-Wallis p-value | Adjusted p-value | Significant Group Differences | Unadjusted p-value | Adjusted p-value |
| --- | --- | --- | --- | --- | --- |
| Age | *<.001* | *<.001* | B-Normal vs. W-Normal  B-High vs W-High  B-Variable vs W-Variable | .64  .006  .13 | .99  .09  .99 |
| Sex | .32 | .99 | N/A | N/A | N/A |
| Education | .32 | .99 | N/A | N/A | N/A |
| Mean Systolic BP | *<.001* | *<.001* | B-Normal vs. W-Normal  B-High vs W-High  B-Variable vs W-Variable | .41  .02  <.001 | .99  .26  <.001 |
| Global Cognition | *<.001* | *<.001* | B-Normal vs. W-Normal  B-High vs W-High  B-Variable vs W-Variable | <.001  <.001  <.001 | <.001  <.001  <.001 |
| Episodic Memory | *<.001* | *<.001* | B-Normal vs. W-Normal  B-High vs W-High  B-Variable vs W-Variable | <.001  .003  <.001 | <.001  .05  <.001 |
| Visuospatial Ability | *<.001* | *<.001* | B-Normal vs. W-Normal  B-High vs W-High  B-Variable vs W-Variable | <.001  <.001  <.001 | <.001  <.001  <.001 |
| Processing Speed | *<.001* | *<.001* | B-Normal vs. W-Normal  B-High vs W-High  B-Variable vs W-Variable | <.001  <.001  <.001 | <.001  <.001  <.001 |
| Semantic Memory | *<.001* | *<.001* | B-Normal vs. W-Normal  B-High vs W-High  B-Variable vs W-Variable | <.001  <.001  <.001 | <.001  <.001  <.001 |
| Working Memory | *<.001* | *<.001* | B-Normal vs. W-Normal  B-High vs W-High  B-Variable vs W-Variable | <.001  <.001  <.001 | <.001  <.001  <.001 |

**Supplemental Table 2**

*Frequency of Antihypertensive/diuretic medication use at baseline and at any point during the study by Race and Blood Pressure group*

|  | | | | | | | |  |
| --- | --- | --- | --- | --- | --- | --- | --- | --- |
| Medication Use | Black | White | Black Normal SBP | Black High SBP | Black Variable SBP | White Normal SBP | White High SBP | White Variable SBP |
| Whole Sample N | 1139 | 1145 | 259 | 351 | 529 | 394 | 332 | 416 |
| Baseline  Medication      (%)  No Medication      (%)  Unknown    (%) | 694 (61%)  183 (16%)  262 (23%) | 639 (56%)  446 (39%)  60 (5%) | 150 (58%)  54 (21%)  55 (21%) | 224 (64%)  49 (14%)  78 (22%) | 320 (60%)  80 (15%)  129 (24%) | 178 (45%)  189 (48%)  29 (7%) | 198 (60%)  119 (36%)  15  (4%) | 262 (63%)  138 (33%)  16  (4%) |
| At Any Point    Medication      (%)  No-Medication      (%)  Unknown    (%) | 812 (71%)  65 (6%)  262 (23%) | 877 (77%)  208 (18%)  60 (5%) | 174 (67%)  30 (12%)  55 (21%) | 254 (72%)  19 (5%)  78 (22%) | 384 (73%)  16  (3%)  129 (24%) | 260 (66%)  107 (27%)  29 (7%) | 267 (80%)  50 (15%)  15 (4%) | 349 (84%)  51 (12%)  16  (4%) |

**Supplemental Table 3**

*Variance of follow-up year as a random slope across global cognition and the five cognitive domains when including and excluding group interaction*

| Cognitive Domain | Model | ID Random Intercept Variance | Follow-up Year Variance | Correlation (Intercept & Slope) | Residual Variance |
| --- | --- | --- | --- | --- | --- |
| Global Cognition | Group Interaction | 0.50 | 0.20 | 0.72 | 0.10 |
|  | No Group Interaction | 0.39 | 0.02 | N/A | 0.21 |
| Episodic Memory | Group Interaction | 0.50 | 0.20 | 0.74 | 0.16 |
|  | No Group Interaction | 0.38 | 0.02 | N/A | 0.27 |
| Visuospatial Abilities | Group Interaction | 0.42 | 0.04 | 0.27 | 0.32 |
|  | No Group Interaction | 0.44 | 0.001 | N/A | 0.34 |
| Processing Speed | Group Interaction | 0.40 | 0.06 | 0.41 | 0.13 |
|  | No Group Interaction | 0.43 | 0.004 | N/A | 0.20 |
| Semantic Memory | Group Interaction | 0.59 | 0.17 | 0.58 | 0.18 |
|  | No Group Interaction | 0.46 | 0.003 | N/A | 0.27 |
| Working Memory | Group Interaction | 0.57 | 0.07 | 0.32 | 0.22 |
|  | No Group Interaction | 0.56 | 0.001 | N/A | 0.26 |
